# A Systematic Review of Temporary Peripheral Nerve Stimulation for Postoperative Pain Management in Orthopedic Surgery

**DOI:** 10.1155/anrp/8477771

**Published:** 2025-12-16

**Authors:** Joshua C. Harris, Chris J. Pierson, Chaitanya Konda, Nitin B. Jain

**Affiliations:** ^1^ College of Medicine, Northeast Ohio Medical University, Rootstown, Ohio, USA, neomed.edu; ^2^ University of Texas Medical Branch Center for Addiction Sciences and Therapeutics, Galveston, Texas, USA; ^3^ Department of Physical Medicine & Rehabilitation, University of Texas Southwestern Medical Center, Dallas, Texas, USA, utsouthwestern.edu; ^4^ Department of Physical Medicine & Rehabilitation, University of Michigan, Ann Arbor, Michigan, USA, umich.edu

## Abstract

**Background/Objectives:**

Over 18 million orthopedic procedures were performed in the United States in 2022 with pharmacological management being the primary mode of analgesia. Percutaneous neuromodulation in the form of temporary peripheral nerve stimulation (tPNS) may provide a new method of postsurgical analgesia devoid of systemic side effects and the potential for substance addiction. tPNS can also be used for chronic pain long after surgery.

**Methods:**

The initial literature search of PubMed/MEDLINE, Web of Science, and Cochrane was conducted on September 21, 2023, and repeated on May 23, 2025. Included studies had patients over 18 years of age with implanted tPNS in the first 2 years following orthopedic surgery of the knee, shoulder, hip, foot, or ankle.

**Results:**

Eleven articles of nine distinct studies were found: eight articles from six randomized trials and three articles from three case series. Seven publications assessed implanted tPNS in the acute perioperative phase, and four publications assessed implanted tPNS for chronic postoperative pain. This systematic review’s aim was to compile current literature on the safety and efficacy of percutaneous tPNS both immediately after orthopedic surgery and for longer‐term postsurgical persistent pain. This collection of evidence suggests using percutaneous tPNS is safe and may reduce pain and postoperative opioid consumption.

**Conclusions:**

This emerging treatment may reduce postoperative pain and opioid consumption, is safe, and warrants further robust trials. Future studies with robust, multimodal treatment designs are needed to specifically delineate the role of tPNS within the context of current pain management.

## 1. Introduction

Chronic musculoskeletal pain is a global health problem with an economic impact second only to cardiovascular disease [1–3] accounting for 1.75 billion people worldwide [4]. Although many types of surgical procedures can improve pain‐related outcomes, the duo of acute and postoperative pain after orthopedic surgery remains a challenge. Among 11 of the most common outpatient surgical procedures, total knee arthroplasty (TKA) is the one with the highest incidence of chronic opioid use [5]. Adequate pain control is crucial, as a high level of postoperative pain is one of the greatest risk factors for the development of chronic pain [6]. Observational studies have found 7.6%–16.3% of patients that report high‐impact chronic pain after total joint arthroplasty [7]. Many pharmacological treatments are available for perioperative and postoperative analgesia; however, these come with the risk of addiction, systemic side effects, or diversion [8].

Temporary peripheral nerve stimulation (tPNS) is an analgesic neuromodulation technique in which a thin wire lead is temporarily inserted under the skin near the target peripheral nerve under ultrasound guidance. This lead is attached to a pulse generator affixed to the patient outside of the skin during treatment. In contrast to treatment with permanently implanted devices, the implanted lead is designed for in‐clinic removal. The lead implant can be completed in‐clinic under local anesthesia without the need for an operative suite. The duration of treatment can vary widely depending on approach, indication, and patient preference. Implanted sensory peripheral nerve stimulation is hypothesized to operate under the gate‐control theory [9] originally proposed by Shealy et al. [10] and Wall and Sweet [11]. The use of multimodal analgesia, with opioid‐sparing options, is a key principle for postoperative pain management. As opioids will continue to have a role in surgical pain management, the overall goal for exploring tPNS is to increase treatment options in the context of multimodal pain management. This has not yet been proposed as a complete alternative to opioid therapy.

A review on the emerging role of tPNS in postoperative analgesia was recently published by Cho et al. [12]. These authors discussed three studies with TKA, one for anterior cruciate ligament reconstruction, one for hallux valgus osteotomy, one for rotator cuff repair, and three for amputation. A recent broad‐reaching review summarized the literature involving tPNS in orthopedic patient populations, including chronic postoperative and nonsurgical pain [13]. The purpose of this review was to expand upon prior work by formally appraising the quality of included studies, describing the direct relationship with opioid medication intake during the acute postoperative phase, and also describing the use of tPNS for chronic postoperative pain.

## 2. Methods

The study was registered on PROSPERO on June 2nd, 2023 (CRD42023428803).

### 2.1. PICO Statement

**(P)** In patients undergoing orthopedic surgery, is **(I)** percutaneous neuromodulation **(C)** in addition to standard of care postoperative pain management **(O)** safe and able to decrease patient‐determined opiate intake?

### 2.2. Search Strategy

The systematic literature review was performed according to the Preferred Reporting Items for Systematic Reviews and Meta‐Analyses (PRISMA) statements [14]. The literature search was conducted on September 21, 2023, and repeated on May 23, 2025, and utilized three databases: PubMed/MEDLINE, Scopus, and Cochrane. EndNote Version 21 was used for reference management. The most recent search found 271 articles. The specific terms and results for each search are described in Appendix A.

After performing the search, titles and abstracts were screened for inclusion. The target patient population included patients 18 years of age or older who had implanted‐lead tPNS treatment following orthopedic surgery of the knee, shoulder, hip, foot, or ankle. Treatments using auricular nerve stimulation, spinal cord stimulation, or electric nerve field stimulation were not included. The reference list of each selected article was then reviewed to search for other applicable articles.

**Figure 1:**
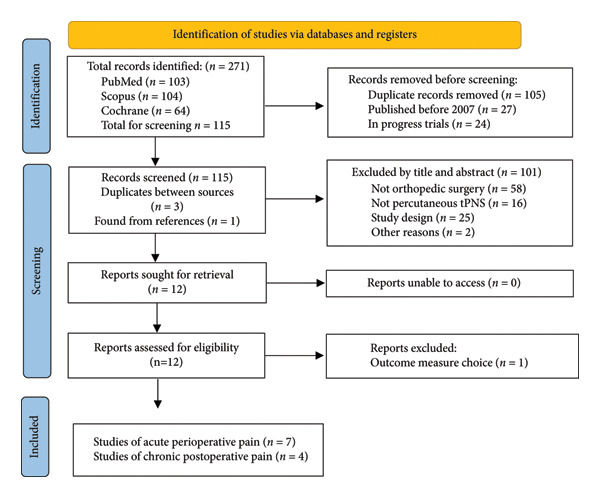
PRISMA flow diagram for article inclusion.

The following study designs were included for data extraction: randomized clinical trials, randomized controlled trials, cohort studies, cross‐sectional studies, and case series. Individual patient case reports were not included. The search strategy is outlined in the PRISMA flow diagram (Figure 1) as instructed by the PRISMA 2020 statement [15].

Inclusion criteria for studies in Figure 1 included publication in the English language, use of a temporarily implanted peripheral nerve stimulator within the first 6 months following orthopedic surgery, oral opioid intake recorded, pain assessed on the Visual Analog Scale (VAS) or Numerical Rating Scale (NRS), and studies published as full reports. Due to advances in the technology of the treatment, studies published prior to 2007 (over 15 years ago) were not included. Older devices had several key differences from the modern devices that preclude a direct comparison. Studies focusing on patients with neuromuscular disease or diagnosed neurological deficits were excluded.

### 2.3. Data Extraction

Following the search and screening, the identified references were distributed to two authors (J.C.H. and C.J.P.). A standardized data extraction spread sheet was created by the research team. Author J.C.H. then used the data extraction spreadsheet to extract data from full‐text articles. An overview of each study is detailed in Table 1, with results and side effects/adverse events detailed in Table 2. Table 3 provides the opioid consumption comparison of the studies involved in this review as well as the author conclusions regarding such.

**Table 1:** Study overview.

First author year PMID	Design	Purpose	*N*	Funding source
Ilfeld 2021 33856424	RT	To assess feasibility and estimate potential benefits and risks of percutaneous PNS for analgesia after a variety of ambulatory surgeries	66	Department of Defense Pain Management Collaboratory, National Center for Complementary and Integrative Health, Office of Behavioral and Social Sciences Research

Ilfeld 2021 34343394	RT (secondary)	To define the analgesic profile beyond the immediate postoperative period and to examine differences between the two different lead placements	61	Department of Defense Pain Management Collaboratory, National Center for Complementary and Integrative Health

Ilfeld 2019 30770421	CS	To evaluate the feasibility of percutaneous PNS following rotator cuff repair in the immediate postoperative period	16	University of California Academic Senate, University of California San Diego Department of Anesthesiology, SPR Therapeutics Inc.

Ilfeld 2019 30160335	RT	To investigate the feasibility of using percutaneous PNS of the femoral nerve to treat pain following ACL reconstruction with a patellar autograft	10	University of California Academic Senate, University of California, San Diego, Department of Anesthesiology, SPR Therapeutics Inc.

Ilfeld 2019 30024078	CS	To determine if using percutaneous PNS is feasible in the immediate perioperative period following TKA. The secondary aims of the study were to investigate the analgesic, opioid‐sparing potential, and impact on functional recovery of percutaneous PNS following TKA relative to published averages.	7	NIH National Institute of Aging, SPR Therapeutics Inc.

Ilfeld 2018 29905630	CS	To demonstrate the feasibility of using percutaneous sciatic nerve PNS to treat postoperative pain following ambulatory foot surgery and provide evidence of analgesic effect	7	University of California Academic Senate, University of California San Diego Department of Anesthesiology, SPR Therapeutics Inc.

Albright‐Trainer 2022 34761694	RT	To evaluate the feasibility of temporary percutaneous PNS for the treatment of acute postamputation pain	16	Hunter Holmes McGuire Research Pilot Grant, SPR Therapeutics Inc.

Wanich 2011 21980881	RT	To evaluate the use of Deepwave as a complementary therapy for efficacy and safety while reducing the severity of acute and chronic pain in patients following TKR surgery, as well as reducing patient opioid use	23	Funding information not found.

Gilmore 2019 30954936	RT	To collect data on the safety and effectiveness of percutaneous PNS for chronic neuropathic pain in amputees.	28	United States Department of Defense Grant, SPR Therapeutics Inc.

Gilmore 2019 31740443	RT (secondary)	To evaluate changes in chronic pain and functional outcomes following amputation up to 12 months after a 60‐day PNS treatment	28	United States Department of Defense Grant, SPR Therapeutics Inc.

Goree 2024 38739062	RT	To evaluate the effect of a 60‐day percutaneous PNS treatment in a multicenter, randomized, double‐blind, placebo‐controlled trial for treating persistent postoperative pain after TKA	53	United States Department of Defense

**Table 2:** Results and side effects/adverse events table.

First author year PMID	Design	Pain level and opioid consumption results	Side effects and adverse events
Ilfeld 2021 33856424	RT	During the first 7 postoperative days, opioid consumption in participants given active stimulation was a median [IQR] of 5 mg [0, 30] vs. 48 mg [25, 90] in patients given sham treatment: ratio of geometric means (97.5% CI) 0.20 (0.07, 0.57), *p* < 0.001. During this same period the average pain intensity in patients given active stimulation was a mean ± SD of 1.1 ± 1.1 vs. 3.1 ± 1.7 in those given sham: difference (97.5% CI) −1.8 (−2.6, −0.9), *p* < 0.001.	One pulse generator stopped functioning the day following surgery and was replaced. One subject with a sciatic lead withdrew on postoperative Day 3 due to unpleasant sensations in the sciatic nerve distribution (he refused to decrease the level of current intensity). One subject developed erythema under the dressing, which resolved following dressing removal (the lead was left in situ and affixed with paper tape by the patient). The leads of two participants fractured during intentional removal.

Ilfeld 2021 34343394	RT (secondary)	For brachial plexus leads, during the first seven postoperative days, pain measured with the numeric rating scale in participants given active stimulation was a median [interquartile range] of 0.8 [0.5, 1.6] vs. 3.2 [2.7, 3.5] in patients given sham (*p* < 0.001). For this same group, opioid consumption in participants given active stimulation was 10 mg [5, 20] vs. 71 mg [35, 125] in patients given sham (*p* = 0.043). For sciatic nerve leads, pain scores for the active treatment group were 0.7 [0, 1.4] vs. 2.8 [1.6, 4.6] in patients given sham (*p* < 0.001). During this same period, participants given active stimulation consumed 5 mg [0, 30] of opioids vs. 40 mg [20, 105] in patients given sham (*p* = 0.004). Treatment effects did not differ statistically between the two locations.	Two lead fractures (3%) were found in the current study without clinical consequences.

Ilfeld 2019 30770421	CS	For subjects with leads targeting the brachial plexus, subsequent average resting and dynamic pain scores POD 1–14 had a median of 1 or less on the NRS; the median dynamic pain score was 3 or less; and opioid requirements averaged less than 1 tablet of oxycodone, 5 mg, daily with active stimulation.	Two leads dislodged during use and four fractured on withdrawal, but no infections, nerve injuries, or adverse sequelae were reported. No infections, nerve injuries, or adverse sequelae resulting from the lead fracture remnant were identified during the final two data collection phone calls on POD 30 or 90.

Ilfeld 2019 30160335	RT	Subjects initially randomized to stimulation (*n* = 5) experienced a slight downward trend in their surgical pain over the 5 min of treatment, while those randomized to sham (*n* = 5) reported a slight upward trend in their surgical pain over the period. The subjects initially receiving sham treatment experienced a similar downward trend in their surgical pain over the second 5‐min crossover period of stimulation. Pain levels for both groups continued to decrease to a mean of 84% of baseline (*n* = 10) during the subsequent 5 min with stimulation. Following this time point, five subjects (50%) requested supplemental opioids, and seven subjects (70%) subsequently initiated the continuous adductor canal nerve block prior to discharge (a mean of 33 min following baseline).	2 subjects reported “poking” in the area of the lead tip beginning on POD 3 and 0, respectively, exclusively when lying supine or standing. This resulted in lead removal at the end of the first post‐op week. One subject reported broken lead outside of the body. This was addressed with a new connecting cable attached to the remaining externalized portion of the lead without any loss of PNS functioning.

Ilfeld 2019 30024078	CS	Four of the seven subjects (57%) had well‐controlled and mild pain and had discontinued opioid use within the first week. One of the four subjects did not use opioids during the entire therapy, while the other three subjects discontinued opioid use on postoperative Days 4, 4, and 6. The median time to opioid cessation across all seven subjects was 6 days.	One subject experienced discomfort at the site of the surface return electrode, which was resolved by moving the surface return electrode to a new location. Another subject experienced bruising around a lead insertion site, which resolved following lead removal without additional treatment. One subject experienced headaches, the cause of which could not be determined. One of 14 leads (7%) was dislodged inadvertently during therapy. Across the seven subjects, 3 of 14 leads (21%) fractured during intentional extraction. The fragments were left in situ and did not produce subsequent complications. No falls, motor blocks, lead infections, or other serious device‐related adverse events were reported.

Ilfeld 2018 29905630	CS	Subjects randomized to stimulation (*n* = 4) experienced a downward trajectory in their pain over the 5 min of treatment, whereas those receiving sham (*n* = 3) reported no such change until their subsequent 5‐min stimulation crossover. During the subsequent 30 min of stimulation, pain scores decreased to 52% of baseline (*n* = 7). Three subjects (43%) used a continuous popliteal nerve block for rescue analgesia during postoperative days 0–3. Overall, resting and dynamic pain scores averaged less than 1 on the numeric rating scale, and opioid use averaged less than 1 tablet (5 mg) daily with active stimulation.	One lead dislodged, 2 fractured during use, and 1 fractured during intentional withdrawal. One adverse event was determined to be unrelated to either the stimulator or continuous popliteal‐sciatic nerve block, with the patient falling on POD 2 without utilizing either intervention the previous 24 h. All lead remnants were left in situ. No lead infections, nerve injuries, or lead fracture remnant sequelae were identified during the final 2 data collection phone calls on POD 30 or 90.

Albright‐Trainer 2022 34761694	RT	By Week 8, the PNS treatment group showed the greatest percent reduction in average phantom (76 vs. 29%) and residual (86 vs. 52%) pain scores compared with the control group. The PNS group ended the 8‐week treatment period with lower scores in all baseline average, worst, and best measures compared with the control group. Though preoperatively the treatment group had a higher percentage of subjects using opioids compared with the control group (36.1 mg vs. 7.2 mg), fewer subjects in the PNS group were using opioids compared with SMT alone at each postoperative time point. Opioid usage increased relative to preoperative levels in the SMT group to 20.3 mg by the end of Week 8 (> 200% increase), while usage decreased relative to preoperative levels to 13.5 mg (> 60% decrease) in the PNS group.	No unanticipated serious study‐related adverse events. No infections occurred during the 8‐week lead implantation period. Two patients required lead re‐implantation due to lead dislodgement. No suspected lead fractures were observed during treatment or lead removal.

Wanich 2011 21980881	RT	There was a significant reduction in the patient’s subjective rating of pain and Visual Analog Scale score in the experimental group (*p* < 0.05), with a trend toward decreased opioid use, but this was not significant (*p* = 0.09).	There were no infections that developed in the perioperative period. There was no evidence of skin irritation or breakdown in any patients. One patient in the experimental group complained of tenderness over the medial PEA, which was replaced with a new PEA with improvement in the discomfort.

Gilmore 2019 30954936	RT	A significantly greater proportion of subjects receiving PNS therapy (58%, 7 of 12, *p* = 0.037) reported ≥ 50% pain relief compared with subjects receiving placebo therapy (14%, 2 of 14) during Weeks 1–4 of the therapy period. Significantly greater proportions of PNS subjects also reported ≥ 50% reductions in pain (*n* = 8/12, 67%, *p* = 0.014) and pain interference (*n* = 8/10, 80%, *p* = 0.003) after 8 weeks of therapy compared with subjects receiving placebo (pain: *n* = 2/14, 14%; pain interference: *n* = 2/13, 15%). 50% (6 of 12) of subjects in the PNS group and 43% (6 of 14) of subjects in the placebo group reduced or stopped opioid and/or non‐opioid medication use.	22 study‐related events were reported in 46% (13 of 28) of subjects who underwent lead implantation, resulting in skin irritation or redness at the lead exit site (7), adhesive return electrode pad site (3) or bandage site (4), pain due to implantation or stimulation (5), pruritus at the pad site (1), pruritus under the supporting belt (1), and fatigue (1). No leads fractured during treatment. In 15% (5 of 34) of leads whose removal was documented, the lead was fractured at the distal tip on removal. There were no serious or unanticipated study‐related adverse events. No leads fractured during treatment. In 15% (5 of 34) of leads whose removal was documented, the lead was fractured at the distal tip on removal.

Gilmore 2019 31740443	RT (secondary)	More participants in Group 1 reported ≥ 50% reductions in average weekly pain at 12 months (67%, 6/9) compared with Group 2 at the end of the placebo period (0%, 0/14, *p* = 0.001). Similarly, 56% (5/9) of participants in Group 1 reported ≥ 50% reductions in pain interference at 12 months, compared with 2/13 (15%, *p* = 0.074) in Group 2 at crossover. Reductions in depression were also statistically significantly greater at 12 months in Group 1 compared with Group 2 at crossover.	22 study‐related events were reported in 46% (13/28) of participants who underwent lead implantation, including 21 mild (96%), 1 moderate (4%), and no severe events (0%). The most common mild events were skin irritation or redness due to the adhesive bandages or pain due to implantation or stimulation, and the one moderate event was pain due to stimulation that was resolved by reprogramming. Five leads were suspected to be fractured during removal. There were no serious or unanticipated study‐related adverse events.

Goree 2024 38739062	RT	Subjects in the PNS group experienced greater pain relief than did those in the placebo group. In the primary endpoint, most subjects (60%; 12/20) in the PNS group were responders (≥ 50% pain relief during Weeks 5–8 of treatment), which was significantly larger than the proportion of responders in the placebo group (24%; 5/21 subjects; *p* = 0.028). Average pain relief across all subjects in the PNS group was significantly greater than in subjects in the placebo group at EOT (54% vs. 26%; *p* = 0.0021). Of the 17 subjects in the PNS group who reported pain medication usage in their baseline diary, most (11/17) reduced pain medication during the primary endpoint period diary as determined by the blinded evaluators. In comparison, ten of 20 subjects in the placebo group reduced pain medication during the primary endpoint period diary. Only a small subset of patients reported baseline opioid use (five in the PNS group and two in the placebo group). Of these subjects, two (one in the PNS group and one in the placebo group) reported a meaningful decrease in opioid use, and one subject (in the placebo group) reported a meaningful increase in opioid use during the primary endpoint period diary.	41 AEs (12 events in the PNS group and 29 events in the placebo group) reported through 1 month after EOT, with 22 subjects reporting ≥ 1 AE (nine subjects in the PNS group and 13 in the placebo group). Mild AEs were reported in 28 of 41 events, moderate AEs in 13 of 41 events, and severe AEs in 0 of 41 events. Dermatologic AEs (e.g., skin irritation due to bandaging) made up most events (37/41; ten events in the PNS group and 27 events in the placebo group). Neurologic AEs (e.g., temporary discomfort) occurred in three events (one event in the PNS group and two events in the placebo group), and one AE was classified as “Other” due to discomfort from an external device component (one event in the PNS group). Across all implanted leads, 97% were removed intact. 2% were removed without the tip of the lead, and 1% were removed with a fracture proximal to the tip of the lead. Leads were left in situ with no additional sequelae. No study‐related AEs were serious or unanticipated.

### 2.4. Risk of Bias Assessment

Two review authors (C.J.P. and J.C.H.) independently assessed the risk of bias for each study using the National Heart, Lung, and Blood Institute (NHLBI) study quality assessment tools to assess the risk of bias for randomized trials (Table 4) or case series (Table 5). Each study was assessed using the applicable questions for each study design [24]. When differences on risk of bias arose, author N.B.J. broke the tie. We established the cutoff score of 80% for bias, with studies under 80% as high risk of bias and studies at or over 80% with low risk of bias. We then used the British Medical Journal system for the Grading of Recommendations Assessment, Development, and Evaluation (GRADE) framework reported in Table 6 [25]. The overall quality of the study was considered “high” if no concerns were noted, “moderate” if there was only one concern, “low” if there were two, and “low to very low” if there were three or more. If we were unable to find an aspect of the GRADE framework, this was noted as “Not Reported.”

**Table 3:** Opioid comparison table.

First author year PMID	Design	Opioid comparison	Conclusions
Ilfeld 2021 33856424	RT	Effects of 14 days of percutaneous peripheral nerve stimulation on opioid consumption (oral morphine equivalents) (immediate‐release oral opioid tablets)	During the first 7 postoperative days, opioid consumption (oral morphine equivalents) in participants receiving active stimulation was a median (interquartile range) of 5 mg (0–30) vs. 48 mg (25–90) in patients given sham (estimated ratio of geometric means, 0.20 [97.5% CI, 0.07 to 0.57]; *p* < 0.001)

Ilfeld 2021 34343394	RT (secondary)	Effects of 14 days of percutaneous peripheral nerve stimulation on opioid consumption (oral morphine equivalents) (immediate‐release oral opioid tablets)	Brachial plexus leads: Participants given active stimulation consumed 10 mg [5, 20] vs. 71 mg [35, 125] in patients given sham (*p* = 0.043) Sciatic Nerve Leads: Participants given active stimulation consumed 5 mg [0, 30] of opioids vs. 40 mg [20, 105] in patients given sham (*p* = 0.004) For nearly all individual days, opioid consumption as well as average and worst pain scores were lower for the stimulation group until lead removal on Day 14

Ilfeld 2019 30770421	CS	Subjects could receive intravenous fentanyl or hydromorphone prior to discharge and/or receive a single injection interscalene nerve block (ropivacaine 0.5%, 20 mL, with epinephrine). Following discharge, subjects were given a prescription for oxycodone 5 mg tablets	Postoperative Days 1–14: opioid requirements averaged less than 1 tablet daily with active stimulation No hypotheses tested

Ilfeld 2019 30160335	RT	Subjects were discharged home with a prescription for oxycodone 5 mg tablets (5–10 mg every 4–6 h to be taken if necessary)	During postoperative Days 1–3, the median daily opioid consumption was less than 1.0 tablet; a clear drop‐off following POD 2 A study suggests that this modality may be effective in providing analgesia and decreasing opioid requirements following anterior cruciate ligament reconstruction with a patellar autograft No hypotheses tested

Ilfeld 2019 30024078	CS	Average daily morphine equivalent dosage [MED] measured for patients postoperatively	Average daily MED during postoperative Days 0–3 was approximately 22 mg Four out of seven subjects had ceased opioid use within the first week (median time to opioid cessation for all subjects was 6 days) A study suggests that perioperative percutaneous PNS may enable earlier opioid cessation No hypotheses tested

Ilfeld 2018 29905630	CS	Subjects were discharged home with a prescription for oxycodone (5 mg tablets) (Lead removal occurred between post‐op Days 14–28)	Opioid use averaged less than 1 tablet daily with active stimulation A study suggests that this modality may decrease opioid requirements following hallux valgus procedures No hypotheses tested

Albright‐Trainer 2022 34761694	RT	Average oral morphine equivalents (in mg) as part of standard medical therapy	A pilot study suggests that PNS is feasible in the acute postoperative period following lower limb amputation and may provide a non‐pharmacologic analgesic therapy that lowers pain scores and reduces opioid consumption No hypotheses tested

Wanich 2011 21980881	RT	Dilaudid (hydromorphone)/bupivacaine epidural patient‐controlled analgesia (PCA) measured in oral morphine equivalents (in mg) for pain management	The Deepwave device appears to be effective in reducing the subjective measures of pain (*p* < 0.05) with a trend toward decreased opioid use in patients following TKR (opioid decrease not significant *p* = 0.09)

Gilmore 2019 30954936	RT	No comparison: patient population using a wide range of opioid and non‐opioid therapies	Percutaneous PNS may provide clinically significant pain relief and functional improvements in patients with neuropathic pain following amputation

Gilmore 2019 31740443	RT	No comparison: patient population using a wide range of opioid and non‐opioid therapies	60‐day percutaneous PNS treatment may provide sustained clinically significant relief of chronic pain following amputation and subsequent improvements in function and depression

Goree 2024 38739062	RT	No comparison for opioid consumption	60‐day percutaneous PNS showed clinically meaningful reductions in pain and improvements in QoL and function that were significantly greater than in the placebo group

Abbreviations: CS = case series, RT = randomized trial.

**Table 4:** Risk of bias for randomized control trials.

First author year PMID	Ilfeld 2021 33856424	Ilfeld 2023 34343394	Ilfeld 2019 30160335	Albright‐Trainer 2022 34761694	Wanich 2011 21980881	Gilmore 2019 30954936	Gilmore 2019 31740443	Goree 2024 38739062
1. Was the study described as randomized, a randomized trial, a randomized clinical trial, or an RCT?	Yes	Yes	Yes	Yes	Yes	Yes	Yes	Yes
2. Was the method of randomization adequate (i.e., use of randomly generated assignment)?	Yes	Yes	Yes	Yes	Yes	Yes	Yes	Yes
3. Was the treatment allocation concealed (so that assignments could not be predicted)?	Yes	Yes	Yes	No	NR	Yes	Yes	Yes
4. Were study participants and providers blinded to treatment group assignment?	Yes	Yes	Yes	No	No	No	No	No
5. Were the people assessing the outcomes blinded to the participants’ group assignments?	Yes	Yes	Yes	No	NR	Yes	Yes	Yes
6. Were the groups similar at baseline on important characteristics that could affect outcomes (e.g., demographics, risk factors, co‐morbid conditions)?	Yes	Yes	Yes	No	Yes	Yes	Yes	Yes
7. Was the overall dropout rate from the study at endpoint 20% or lower of the number allocated to treatment?	Yes	Yes	Yes	No	Yes	Yes	No	No
8. Was the differential dropout rate (between treatment groups) at endpoint 15 percentage points or lower?	Yes	Yes	Yes	Yes	Yes	Yes	No	Yes
9. Was there high adherence to the intervention protocols for each treatment group?	Yes	Yes	Yes	Yes	NR	Yes	Yes	NR
10. Were other interventions avoided or similar in the groups (e.g., similar background treatments)?	Yes	Yes	Yes	Yes	Yes	NR	NR	Yes
11. Were outcomes assessed using valid and reliable measures, implemented consistently across all study participants?	Yes	Yes	Yes	Yes	Yes	Yes	Yes	Yes
12. Did the authors report that the sample size was sufficiently large to be able to detect a difference in the main outcome between groups with at least 80% power?	No	No	NA	No	No	No	No	No
13. Were outcomes reported or subgroups analyzed prespecified (i.e., identified before analyses were conducted)?	Yes	NR	NA	NA	NR	Yes	Yes	Yes
14. Were all randomized participants analyzed in the group to which they were originally assigned, that is, did they use an intention‐to‐treat analysis?	Yes	Yes	Yes	Yes	Yes	Yes	Yes	Yes
Total Score	13/14	12/13	12/12	7/13	8/10	11/13	9/13	10/13
High risk of bias if under 80%, low risk of bias if 80% or more	Low risk, 93%	Low risk, 92%	Low risk, 100%	High risk, 54%	Low risk, 80%	Low risk, 84%	High risk, 69%	High risk, 77%

Abbreviation: NR = not reported.

**Table 5:** Risk of bias for case series.

First author year PMID	Ilfeld 2019 30024078	Ilfeld 2019 30770421	Ilfeld 2018 29905630
1. Was the study question or objective clearly stated?	Yes	Yes	Yes
2. Was the study population clearly and fully described, including a case definition?	Yes	Yes	Yes
3. Were the cases consecutive?	NA	NA	NA
4. Were the subjects comparable? (Look at within that group)	Yes	Yes	No
5. Was the intervention clearly described?	Yes	Yes	Yes
6. Were the outcome measures clearly defined, valid, reliable, and implemented consistently across all study participants?	Yes	Yes	Yes
7. Was the length of follow‐up adequate?	Yes	Yes	Yes
8. Were the statistical methods well‐described? (Depends on how it was presented)	NA	NA	NA
9. Were the results well‐described?	Yes	Yes	Yes
Total Score	7/7	7/7	6/7
High risk of bias if under 80%, low risk of bias if 80% or more	Low risk, 100%	Low risk, 100%	Low risk, 85.7%

**Table 6:** GRADE framework.

First author year PMID	Ilfeld 2023 34343394	Ilfeld 2021 33856424	Ilfeld 2019 30770421	Ilfeld 2019 30160335	Ilfeld 2019 30024078	Ilfeld 2018 29905630	Albright‐Trainer 2022 34761694	Wanich 2011 21980881	Gilmore 2019 30954936	Gilmore 2019 31740443	Goree 2024 38739062
Design	RT	RT	CS	RT	CS	CS	RT	RT	RT	RT	RT
Serious Inconsistency	No	No	NR	NR	NR	No	NR	No	No	No	No
Serious Indirectness	No	No	No	No	No	No	No	No	No	No	No
Serious Imprecision	No	No	NR	NR	NR	NR	Possible	Possible	No	Possible	No
Publication Bias	None	None	None	None	None	None	None	None	None	None	None
Risk of Bias	Low	Low	Low	Low	Low	Low	High	Low	Low	High	High
Total Score	5/5	5/5	3/3	3/3	3/3	4/4	2/4	4/5	5/5	3/5	4/5
Overall Quality	High	High	High	High	High	High	Low	Moderate	High	Low	Moderate

Abbreviations: CS = case series, NR = not reported, RT = randomized trial.

### 2.5. Outcome Measures

Included studies assessed oral opioid consumption as an outcome measure following orthopedic surgery (Table 2). Different authors used different methodologies for this; however, most studies were from the same research group and thus were reported in the same way.

## 3. Results

We included 11 total analyses from nine distinct studies. Seven assessed tPNS for acute postoperative pain and four for chronic postoperative pain.

### 3.1. Randomized Trials

A single multisite randomized controlled study was found assessing the use of tPNS following arthroscopic surgery, implanted adjacent to either the brachial plexus or the sciatic nerve [16]. This study (*n* = 66) had an overall high quality with a low risk of bias. The median 7‐day opioid consumption was 5 mg in the treatment group and 48 mg in the sham group (*p* = 0.001) and the mean “average” daily pain intensity was 1.1/10 in the treatment group compared to 3.1/10 in the sham group. It was concluded that tPNS was overall better for pain management than sham for the first 7 days [16]. A secondary analysis of this trial compared the effect of tPNS between surgeries around the brachial plexus and surgeries around the sciatic nerve. This trial saw an average difference in brachial plexus tPNS group versus control of 10 mg opioid consumption versus 71 mg. This trial saw an average difference in the sciatic nerve tPNS group versus the control of 5 mg opioid consumption versus 40 mg. Brachial plexus surgery patients reported a median pain score of 0.8/10 versus 3.2/10 of control patients, while sciatic nerve patients had a difference of 0.7/10 versus 2.8/10 between tPNS and control groups.

A single‐site randomized clinical trial evaluated the feasibility and safety of tPNS for TKA postoperative pain management. This study had a low risk of bias and moderate quality within the GRADE review system. There was a possible risk of serious imprecision due to a lack of explanation of statistical analysis and ambiguous visual representation of data. The trial results do support the feasibility and justification for further study with a larger study size [21].

A single‐site randomized controlled feasibility trial was found assessing the effectiveness of percutaneous tPNS to improve postamputation pain [26]. The authors described this as a randomized controlled pilot study aimed to assess feasibility, and did not purport this as a definitive clinical trial. Evaluated as a controlled trial, this was a low‐quality study with a high risk of bias. We were unable to assess this as a case series, as patients were randomized, and a direct comparison was made between the treatment group and the control group. It was found that the tPNS treatment group had greater reductions in average phantom limb pain, residual limb pain, and daily opioid consumption. It was also found that there were fewer participants in the tPNS group taking opiates 3 months postamputation [26].

A single‐site proof‐of‐concept trial (*n* = 10) was found assessing the use of tPNS to the femoral nerve following anterior cruciate ligament reconstruction surgery [19]. This study used a randomized design but was not intended to assess definitive clinically significant results. This high‐quality and low‐risk‐of‐bias study found the median opioid consumption was less than 5 mg oxycodone per day. Inferential statistics were not applied, but the outcome measure results did not appear to be clinically significant [19].

A multicenter, randomized, double‐blind, placebo‐controlled partial‐crossover trial demonstrates that percutaneous PNS therapy may provide enduring clinically significant pain relief and improve disability in patients with chronic neuropathic postamputation pain [22]. Authors found that subjects receiving PNS therapy (58%, 7 of 12, *p* = 0.037) reported ≥ 50% pain relief compared with subjects receiving placebo therapy by the primary endpoint of the study at 4 weeks. This was followed by a crossover from the initial placebo group that resulted in improvement of phantom limb pain but not residual limb pain. This study was assessed and GRADED as high‐quality with a low risk of bias. A secondary analysis from this trial reported on the long‐term follow‐up outcomes. The study found statistically and clinically significant reduction in pain relief and pain interference, but the authors acknowledge the inherent effects of participant withdrawal on the observed effects. It was found that this study design was subject to possible imprecision within the scope of the study and statistical analysis. It was also noted that this study was at high risk of bias due to high overall dropout rates and differential dropout rates between study groups.

A multicenter, double‐blind, placebo‐controlled, randomized trial examined postoperative pain management after TKA with implanted tPNS. This study was appraised as being of moderate quality given the high risk of bias due to the methodological limitations and lack of explicit statistical justification of sample size (*n* = 53). This study reports greater pain relief at the primary endpoint with tPNS, further supporting usage within the context of orthopedic surgery pain management [23].

### 3.2. Case‐Series Studies

A single‐site feasibility study was found assessing the use of tPNS to the femoral and sciatic nerves following TKA [18]. This high‐quality study had an overall low risk of bias. The study suggests earlier opioid cessation following TKA. This study (*n* = 7) found six of seven subjects having average daily pain scores for the first 2 weeks under 4/10 NRS [18].

A single‐site feasibility study (*n* = 16) was found assessing the use of tPNS to the suprascapular nerve and brachial plexus following rotator cuff repair surgery [17]. This was a high quality and low risk of bias case series. Although a randomized, double‐blinded, crossover design was used for pain in the recovery room, the authors reported the sample size was too small to use inferential statistics. However, subjects appeared to demonstrate adequate pain control during POD 1–14, requiring less than 1 tablet of 5 mg oxycodone per day with a median NRS score of 1/10 or less [17].

A single‐site feasibility study (*n* = 7) was found assessing the use of tPNS to the sciatic nerve following ambulatory foot surgery [20]. This was a high quality and low risk of bias case series. Although a randomized, double‐blinded, crossover design was used for pain in the recovery room, the authors reported the sample size was too small for inferential statistics. Subjects appeared to demonstrate adequate pain control during postoperative Days 1–90, requiring less than 1 tablet of 5 mg oxycodone per day with a median NRS score (at rest) of 1 or less besides postoperative Day 2 [20].

## 4. Discussion

The goal of this review was to summarize and appraise the current evidence on the use of tPNS for both acute and chronic postoperative pain following orthopedic surgery. Orthopedic surgery is one of the fastest‐growing surgical specialties, with a total of 18.56 million procedures performed in the United States alone in 2022 [27]. Ten publications were found with the search that fit our prespecified inclusion criteria for analysis, and an eleventh was found by searching the references. Three used a case‐series study design, and seven used a randomized trial design. When compared directly to a placebo or standard of care, these generally found analgesic success. No study reported worse outcomes with the use of tPNS. Only one trial directly assessed noninferiority, which found tPNS to be noninferior [16].

Of the publications surveyed, the adverse event profile was acceptable in each study, with no serious adverse events (such as death, systemic infection, or hospitalization) attributed to the stimulation treatment. The most common minor adverse event was a lead fracture during lead removal, but no studies reported clinically important sequelae as a result. Although some studies were more focused on technical feasibility, all assessed postoperative opiate intake or pain level with the use of tPNS.

Recent advances in technology have addressed the adverse effects of previous tPNS techniques of lead fracture and subsequent complications. With concomitant advances, more recent studies have explored the feasibility of tPNS for non‐opioid analgesia for orthopedic surgical pain management. Lead fragmentation has historically been a concern, with a 2007 review reporting lead failure rates of 56%–80% within three months of implantation [28]. In contrast, a 2023 review found a 6.25% rate of retained lead fragments following removal [29].

Strength throughout many of these projects was demonstrated in the highly specific patient inclusion and exclusion criteria. This was often made robust by recruiting patients undergoing specific orthopedic surgical procedures. Another strength of this body of literature was the breadth of orthopedic surgery procedures studied. This body of evidence is representative of the most common orthopedic surgery procedures performed in the United States [5]. There was also insignificant variation in the treatment techniques used, with most of the intervention devices coming from a single company and using the same parameters for acute or for chronic pain.

The current body of research is limited by the lack of diversity of research environments. Most of the ten publications included in this review were published by lead authors from a single center from 2017 to 2024. Research environment diversity may improve in the future, as one multicenter randomized, controlled trial demonstrated that this research technique is feasible at several different institutions across the United States [16]. Many of the included studies did not report a power calculation, as they were small feasibility studies with 11 or fewer participants. Additionally, among the controlled trials, there are variable statistical methods.

In addition to the published manuscripts described, a search on clinicaltrials.gov found two more relevant ongoing projects. A 60‐patient interventional study evaluating the efficacy of percutaneous neuromodulation to the femoral nerve following anterior cruciate ligament reconstruction was also started in 2022 (NCT05606250). A 64‐patient interventional study evaluating femoral nerve neuromodulation is ongoing to answer if this improves analgesic quality and short‐term functional recovery following TKA (NCT05971095).

## 5. Conclusions

Multiple feasibility studies and nascent randomized trials were found assessing the safety and feasibility of tPNS for patients undergoing orthopedic surgery. Treatment with tPNS may reduce total opioid consumption and duration of opioid usage, although larger and more robust trials are warranted to further assess treatment efficacy of tPNS. tPNS treatment is emerging as a safe and effective technique that could prove to be a meaningful synergistic modulator to the opioid analgesics regularly prescribed following surgery.

## Conflicts of Interest

The authors declare no conflicts of interest.

## Author Contributions

Joshua C. Harris: This author performed the search, assessed risk of bias, created the tables, extracted data from each article, and contributed to manuscript writing.

Chris J. Pierson: This author developed the idea for the manuscript, performed the search, assessed the risk of bias, and contributed to manuscript writing.

Chaitanya Konda: This author provided clinical insight and contributed to manuscript writing.

Nitin B. Jain: This author advised Joshua and Chris on the systematic review process, assessed risk of bias, and contributed to manuscript writing.

## Funding

This review has no funding sources to declare.

## Data Availability

Data collection templates and data extraction forms are available upon request to the corresponding author.

## Appendix A Search Strategy

First Search Terms: Percutaneous neuromodulation (PubMed *n* = 65) (Scopus *n* = 72) (Cochrane *n* = 58).

Contains TITLE only.

Second Search Terms: Peripheral Nerve Neurostimulation (PubMed *n* = 12) (Scopus *n* = 15) (Cochrane *n* = 2) Contains TITLE only.

Third Search Terms: Implanted peripheral nerve stimulation (PubMed *n* = 13) (Scopus *n* = 15) (Cochrane *n* = 4) Contains TITLE only.

Fourth Search Terms: Percutaneous Neurostimulation (Article Title) and orthopedic surgery (all fields) (PubMed *n* = 13) (Scopus *n* = 4) (Cochrane *n* = 0).
